# Thrombospondin-1 expression and modulation of Wnt and hippo signaling pathways during the early phase of *Trypanosoma cruzi* infection of heart endothelial cells

**DOI:** 10.1371/journal.pntd.0010074

**Published:** 2022-01-05

**Authors:** Ashutosh Arun, Kayla J. Rayford, Ayorinde Cooley, Tanu Rana, Girish Rachakonda, Fernando Villalta, Siddharth Pratap, Maria F. Lima, Nader Sheibani, Pius N. Nde

**Affiliations:** 1 Department of Microbiology, Immunology and Physiology, Meharry Medical College, Nashville, Tennessee, United States of America; 2 Department of Professional Medical Education and Molecular Biology Core Facility, Meharry Medical College, Nashville, Tennessee, United States of America; 3 School of Graduate Studies and Research, Meharry Medical College, Nashville, Tennessee, United States of America; 4 Department of Molecular and Cellular and Biomedical Sciences, School of Medicine, The City College of New York, New York, United States of America; 5 Department of Ophthalmology and Visual Sciences, Biomedical Engineering, and Cell and Regenerative Biology, University of Wisconsin School of Medicine and Public Health, Madison, Wisconsin, United States of America; University of California San Diego, UNITED STATES

## Abstract

The protozoan parasite, *Trypanosoma cruzi*, causes severe morbidity and mortality in afflicted individuals. Approximately 30% of *T*. *cruzi* infected individuals present with cardiac pathology. The invasive forms of the parasite are carried in the vascular system to infect other cells of the body. During transportation, the molecular mechanisms by which the parasite signals and interact with host endothelial cells (EC) especially heart endothelium is currently unknown. The parasite increases host thrombospondin-1 (TSP1) expression and activates the Wnt/β-catenin and hippo signaling pathways during the early phase of infection. The links between TSP1 and activation of the signaling pathways and their impact on parasite infectivity during the early phase of infection remain unknown. To elucidate the significance of TSP1 function in YAP/β-catenin colocalization and how they impact parasite infectivity during the early phase of infection, we challenged mouse heart endothelial cells (MHEC) from wild type (WT) and TSP1 knockout mice with *T*. *cruzi* and evaluated Wnt signaling, YAP/β-catenin crosstalk, and how they affect parasite infection. We found that in the absence of TSP1, the parasite induced the expression of Wnt-5a to a maximum at 2 h (1.73±0.13), P< 0.001 and enhanced the level of phosphorylated glycogen synthase kinase 3β at the same time point (2.99±0.24), P<0.001. In WT MHEC, the levels of Wnt-5a were toned down and the level of p-GSK-3β was lowest at 2 h (0.47±0.06), P< 0.01 compared to uninfected control. This was accompanied by a continuous significant increase in the nuclear colocalization of β-catenin/YAP in TSP1 KO MHEC with a maximum Pearson correlation coefficient of (0.67±0.02), P< 0.05 at 6 h. In WT MHEC, the nuclear colocalization of β-catenin/YAP remained steady and showed a reduction at 6 h (0.29±0.007), P< 0.05. These results indicate that TSP1 plays an important role in regulating β-catenin/YAP colocalization during the early phase of *T*. *cruzi* infection. Importantly, dysregulation of this crosstalk by pre-incubation of WT MHEC with a β-catenin inhibitor, *endo*-IWR 1, dramatically reduced the level of infection of WT MHEC. Parasite infectivity of inhibitor treated WT MHEC was similar to the level of infection of TSP1 KO MHEC. These results indicate that the β-catenin pathway induced by the parasite and regulated by TSP1 during the early phase of *T*. *cruzi* infection is an important potential therapeutic target, which can be explored for the prophylactic prevention of *T*. *cruzi* infection.

## Author summary

*Trypanosoma cruzi*, the causative agent of Chagas disease, is now considered a global health threat in all industrialized regions of the world. About 30% of infected individuals will develop cardiac pathology. The molecular mechanisms by which the parasite signals and infect host cells to cause pathology remain to be elucidated. We showed that host thrombospondin-1 (TSP1) is essential for *T*. *cruzi* infection since cells deficient in TSP1 are poorly infected by the parasite. In this study, we challenged mouse heart endothelial cells from wild type and TSP1 KO mice and evaluated the importance of TSP1 in Wnt/β-catenin signaling, β-catenin/YAP colocalization and how it can be exploited to regulate parasite infectivity. The parasite activated Wnt/β-catenin signaling pathway and nuclear β-catenin/YAP colocalization. In TSP1 KO MHEC (absence of TSP1), the parasite induced a continuous increase in nuclear translocation and colocalization of β-catenin/YAP. These results suggest that during the early phase of *T*. *cruzi* infection, TSP1 regulates β-catenin/YAP interaction. Inhibition of the Wnt/β-catenin pathway in WT MHEC led to a significant reduction of parasite infectivity. The level of parasite infection of the pretreated WT cells was as low as that of TSP1 KO MHEC. Our studies show that the β-catenin pathway is an important therapeutic target for *T*. *cruzi* infection.

## Introduction

*Trypanosoma cruzi*, the causative agent of Chagas heart disease is an obligate intracellular parasite that can infect all nucleated cells of the body. The disease, which was originally endemic in Mexico and other Latin American countries, is now present in all economically advanced countries of the world including the United States in particular due to modern globalization making it a new global health threat [1–5]. The disease originally transmitted through the kissing bug vector, *Triatoma infestans*, has now been shown to be transmitted by several other modes including the use of contaminated blood/blood products, needles, use of contaminated organs during organ transplant and transmission through the oral route during consumption of contaminated foods or drinks [6].

Vertical transmission from mother-to-child has now become a new research and public health policy challenge especially as there is a substantial population of *T*. *cruzi*-infected women of childbearing age and congenitally infected infants among the Latin American migrants [7,8]. In addition to vertical transmission, autochthonous vector transmissions have been reported in the emerging infection regions of the world including the United States [9,10]. During the process of cellular infection, invasive *T*. *cruzi* trypomastigotes infect host cells and transform to replicative amastigotes within the infected cells. The amastigotes multiply and transform to invasive trypomastigotes, which are released to infect other cells. Some of the released trypomastigotes infect neighboring cells while others are transported in blood through the host’s vascular system to infect cells in distant parts of the body. During parasite transportation in the blood, invasive trypomastigotes interact with and potentially infect endothelial cells lining the internal surface of the vascular wall through which blood is transported.

Endothelial cells lining the inner surface of the vascular system are surrounded by the extracellular matrix proteins, which interact with matricellular proteins. Matricellular proteins are extracellular matrix (ECM) proteins that interact with cells and other ECM components to regulate cellular behavior and ECM organization but are not part of the structural elements of the ECM [11–14]. One of the matricellular proteins, thrombospondin-1 (TSP1) is a complex homotrimeric secreted glycoprotein belonging to the group A subfamily of five TSP family members [15]. TSP1 interacts with several extracellular molecules including, matrix regulating enzymes, glycosaminoglycans, growth factors and diverse cellular receptors among others, thereby having an important role in tissue and cellular homeostasis [13,16–19]. TSP1 has been reported to be very essential in cardiovascular health since it plays important roles in the function of vascular cells including vascular smooth muscle cells, inflammatory cells, fibroblasts and endothelial cells [20] which is of interest in this manuscript.

We showed that TSP1 plays a very important role in the process of host cell infection by *T*. *cruzi*. The parasite induces the expression of TSP1 in host cells, including primary human coronary artery smooth muscle cells, to facilitate cellular infection [21]. However, the changes in cellular homeostasis caused by the parasite induced increase in TSP1 expression remains unknown. Furthermore, we showed that the expressed host TSP1 interacts with *T*. *cruzi* calreticulin (TcCRT) on the surface of the parasite to facilitate cellular infection, which was inhibited in the presence of TcCRT monovalent Fab antibody and in the absence of TSP1 [22]. We also showed that higher levels of host TSP1 induced by the parasite dysregulated the levels of phosphorylated proteins and cellular signaling in the challenged cells [23]. We showed that overexpression of TSP1 increased *T*. *cruzi* cellular infection while RNAi knockdown or absence of TSP1 led to a significant decrease in cellular infection [21,22].

The Wnt signal transduction cascade plays important roles in cardiac pathophysiology and its dysregulation can lead to cardiac dysfunction, such as cardiac hypertrophy, fibrosis, arrhythmias, and infarction [24–26]. In the classical canonical Wnt signaling pathway, when a Wnt ligand is absent, the Wnt-off state, a destruction protein complex is formed. The complex is composed of a scaffolding protein axin, adenomatous polyposis coli (APC) tumor suppressor protein, glycogen synthase kinase 3β (GSK3β), and casein kinase 1 (CK1). The GSK3β component of the complex phosphorylates β-catenin at amino acid residues Ser33, Ser37 and Thr41 at the amino-terminal end of β-catenin, while the CK1 component phosphorylates β-catenin at the amino acid residues Ser45 [27,28]. This phosphorylation pattern on β-catenin signals the ubiquitination and subsequent proteasomal degradation of the post-translationally modified β-catenin. Consequently, β-catenin does not translocate to the nucleus and the repressor complex composed of T-cell specific factor (TCF)/lymphoid enhancer-binding factor (LEF) and transducing-like enhancer protein (TLE)/Groucho bind to specific regions of cellular DNA to block the transcription of Wnt responsive genes [29–31].

When a Wnt ligand is present, the Wnt-on state, it binds to one of several Frizzled (Fz) receptors and the low-density lipoprotein (LDL) receptor–related protein (LRP)5/6 coreceptors to activate the canonical Wnt signaling pathway. The cytoplasmic tail of LRP is phosphorylated by GSK3β and CK1 and becomes activated [32,33]. The activated transmembrane co-receptors (Fz and LRP) recruit the cytoplasmic protein dishevelled (Dvl) to the cell membrane, thus, sequestering the rate-limiting components axin and GSK3β from forming the destruction complex [34]. The hypo-phosphorylated/stabilized form of cytosolic β-catenin accumulates and is translocated to the nucleus to displace the inhibitor Groucho, interact with the TCF/LEF family of transcription factors and activate transcription of Wnt responsive genes [27,30,31,35].

The Hippo signaling pathway, which was originally identified in *Drosophila melanogaster* has now been extensively reported in mammalian cells. This pathway plays an important evolutionarily role in the regulation of organ size, cell proliferation, and apoptosis [36,37]. In mammals, when the canonical Hippo signaling cascade is turned on, serine residues (S127 and 381) in the Hippo effector Yes-associated protein (YAP) and serine residues (S89 and 311) in the transcriptional coactivator with PDZ-binding motif (TAZ) are phosphorylated initially by activated LATS1/2—MOB1A/B complex followed by CK1 kinase. The phosphorylation pattern on the YAP/TAZ effector molecules is a signal for interaction with 14-3-3 for cytoplasmic retention or ubiquitination-mediated degradation [38–40]. In contrast, when the pathway is turned off, YAP/TAZ are not phosphorylated. They are translocated into the nucleus where they interact with TEA domain family members/transcription enhancer factor (TEADs/TEF) family of transcriptional co-activators to promote transcription of downstream genes including TSP1, which plays an important role in cell growth and proliferation [38,41].

Recently, multiple layers of complex crosstalk between Wnt signaling and YAP/TAZ activity have been reported in several different contexts, especially in linking fibrosis and cancer research [42]. YAP/TAZ cooperation with β-catenin has been suggested to be essential for the induction of gastrointestinal tumorigenesis [43,44]. Furthermore, transgene YAP overexpression in intestinal epithelial cells increased both β-catenin expression and their downstream transcriptional targets including cyclin D1 [45]. We showed that *T*. *cruzi* upregulates the expression of TSP1, which is downstream of hippo signaling during the early phase of infection. However, the crosstalk between Hippo and Wnt signaling pathways and the role that TSP1 plays in that crosstalk during the early phase of *T*. *cruzi* infectivity remains unknown.

We hypothesize that during the early phase of molecular interaction between *T*. *cruzi* and heart endothelial cells, TSP1 plays an important role in the nuclear colocalization/crosstalk between the Hippo effector molecule YAP, and the Wnt effector molecule β-catenin through an unknown mechanism. To delineate the significance of TSP1 expression on YAP and β-catenin nuclear colocalization during the early phase of infection, we challenged MHEC from wild type (WT) and TSP1 knockout (TSP1 KO) mice with *T*. *cruzi*. We evaluated the kinetics of Wnt signaling pathway-associated proteins, nuclear translocation of hippo effector molecule YAP, and its nuclear colocalization with β-catenin. Here we show that in the absence of TSP1 (TSP1 KO MHEC) the parasite induces an increase in the levels of β-catenin in the total cell lysate and in the nuclear compartment. This was accompanied by a significant increase in the nuclear translocation of YAP and its colocalization with β-catenin. In contrast, in the presence of TSP1, WT MHEC, we observed a decrease in the levels of nuclear translocated β-catenin and a consequent significant decrease in YAP/β-catenin nuclear colocalization.

To further evaluate the significance of these effector molecules in the process of *T*. *cruzi* cellular infection, we preincubated WT MHEC with 20 μM *endo*-IWR 1 inhibitor, which increases axin2 protein expression, stabilizes the destruction complex and promotes β-catenin phosphorylation. The pretreated cells were then challenged with the parasite to evaluate alterations in infectivity. We observed that inhibition of β-catenin pathway with *endo*-IWR 1 significantly reduced the level of *T*. *cruzi* infectivity. The level of *T*. *cruzi* infection in the *endo*-IWR1 inhibitor treated WT MHEC was as low as the level of *T*. *cruzi* infectivity of TSP1 KO MHEC. Our data advances the understanding of the molecular interactions occurring between endothelial cells and *T*. *cruzi* in the presence and absence of TSP1 during the early phase of *T*. *cruzi* infection and pathogenesis. Furthermore, our data also show that inhibition of the β-catenin pathway is a potential prophylactic/therapeutic target for the prevention of *T*. *cruzi* infection.

## Methods

### Ethics statement

The animal studies were carried out in accordance with the protocol number 150514PN093 approved by the Institutional Animal Care and Use Committee (IACUC) of Meharry Medical College.

### Generation and culture of Mouse Heart Endothelia Cells (MHEC)

MHEC were isolated and maintained as previously described for retinal EC [46]. Briefly, hearts were harvested from 4-week-old WT or TSP1 KO Immorto mice as detailed [46]. Hearts (from 3–4 mice) were pooled together and minced in DMEM in a 60 mm tissue culture dish using sterile razor blades followed by digestion in 5 ml of collagenase type I (1 mg/ml in serum free DMEM, Worthington, Lakewood, NJ) for 30–45 min at 37°C. DMEM containing 10% FBS was added and cells were pelleted. The cellular digests were filtered through a double layer of sterile 40 μm nylon mesh (Sefar America Inc., Fisher Scientific, Hanover Park, IL), and cells pelleted at 400x g for 10 min. Cells were washed twice with DMEM containing 10% FBS, resuspended in 1.5 ml of wash medium, and incubated with sheep anti-rat magnetic beads pre-coated with anti-PECAM-1 as described [46]. After affinity binding, magnetic beads were washed extensively with wash medium and bound cells in endothelial cell growth medium were plated into a single well of a 24 well plate pre-coated with 2 μg/ml of human fibronectin (BD Biosciences, Bedford, MA). Endothelial cells were grown in DMEM containing 10% FBS, 2 mM L-glutamine, 2 mM sodium pyruvate, 20 mM HEPES, 1% non-essential amino acids, 100 μg/ml streptomycin, 100 U/ml penicillin, freshly added heparin at 55 U/ml (Sigma, St. Louis, MO), endothelial growth supplement 100 μg/ml (Sigma, St. Louis, MO), and murine recombinant interferon-γ (R & D, Minneapolis, MN) at 44 units/ml. Cells were maintained at 33°C with 5% CO_2_. The beads either fell off, get digested or diluted out as we expand the initial EC isolation. To confirm that these cells are EC, we examined the expression of two endothelial cell specific markers, PECAM-1 and VE-cadherin, by FACS analysis. Nearly 100% of wild type and TSP1 KO MHEC expressed high levels of these markers on their surface. Early passage cultures of WT and TSP1 KO MHEC were used in our studies.

### Rat heart myocyte culture

Rat heart myoblasts (RHM) were maintained in DMEM containing glutamax (Life Technologies), 10% heat inactivated fetal bovine serum (Life Technologies), 1% Penicillin/Streptomycin (Life Technologies), 1% Non-essential amino acid and multivitamin (Life Technologies). RHM were grown in humidified tissue culture incubator at 37°C and 5% CO_2_.

### Invasive *T*. *cruzi* trypomastigote generation and infection assays

Rat heart myoblasts (RHM) used for the generation of invasive *T*. *cruzi* trypomastigotes clone MMC 20A (Tulahuen strain) were maintained in DMEM containing glutamax (Life Technologies), 10% heat inactivated fetal bovine serum (Life Technologies), 1% Penicillin/Streptomycin (Life Technologies), 1% Non-essential amino acid and multivitamin (Life Technologies) at 37°C and 5% CO_2_. RHM monolayers (80% confluency) infected with *T*. *cruzi* trypomastigotes clone MMC 20A (Tulahuen strain) were fed with fresh complete medium daily. Released pure population of highly invasive *T*. *cruzi* trypomastigotes were harvested as previously described [47–49]. The parasites were washed in Hank’s Balanced Salt Solution (HBSS) and resuspended in MHEC growth medium without supplement at a concentration of 1x10^7^ parasites/ml for use in our experiments. Transgenic *T*. *cruzi* trypomastigotes clone MMC 20A (Tulahuen strain) overexpressing green fluorescent protein (GFP–*T*. *cruzi*) were similarly generated [22,50]. For infection assays, MHEC monolayers (approximately 85% confluency) were starved in medium without supplements followed by addition of invasive *T*. *cruzi* trypomastigotes at a ratio of 10 parasites per cell. Cells challenged with the parasites were incubated for different time points 1, 2, 3 and 6 hours, respectively. At the end of each time point, parasites were washed with 1x DPBS (without calcium/magnesium) and the cells were either stored at -80°C or processed immediately for western blot analysis. The biological replicates of each time point were prepared in independent T75 flasks for use in the western blotting experiments or independent sets of 6 well culture plates containing coverslip coated with 1% gelatin for use in confocal immunofluorescence microscopy assays. Mock-infected (medium only) MHEC were used as control for each time point. For fluorescence microscopy, infection assays were similarly performed on 6 well plastic culture plates. NucBlue Live ReadyProbes Reagent (Thermofisher Cat #R37605) was added to the cells as a nuclear stain, 2 drops/mL of media, for 20 minutes and washed, essentially as described by manufacturer. Cells were also incubated with CellMask Orange Actin Tracking Stain (Thermofisher Cat #A57247) at 1:1000 dilution for 40 minutes to stain actin and washed essentially as described by the manufacturer. The stained cells were visualized using the Keyence All-in-One Fluorescence Microscope BZ-X710 and analyzed with BZ-X Analyzer software.

### Immunoblotting assays

MHEC (WT or TSP1 KO) monolayers were challenged with *T*. *cruzi* trypomastigotes at the different time points (1, 2, 3 and 6 hours) and the parasites were washed off with DPBS (without calcium or magnesium). Control and parasite challenged cell monolayers were lysed in RIPA buffer (Life Technologies) containing protease inhibitor cocktail set III at 1:100 (Calbiochem, Gibbstown, NJ, USA), phosphatase inhibitor cocktails 2 and 3 at 1:100 each (Sigma Aldrich, St. Louis, MO, USA). Whole cell lysates (20 μg/well) were separated by SDS-PAGE using 4–15% gradient polyacrylamide gels and transferred onto nitrocellulose membranes (Life Technologies). The membranes were incubated in Intercept TBS Blocking Buffer (LI-COR Biosciences, Lincoln, Nebraska, USA) followed by incubation with appropriate primary antibody diluted at 1:1000 at 4°C overnight on a shaker; mouse anti-Wnt-5a monoclonal antibody (Santa Cruz Biotechnology, Dallas, Texas, USA; cat no. sc-365370), mouse anti-GAPDH monoclonal antibody (Santa Cruz Biotechnology, cat no sc-47724), rabbit anti-β-Catenin antibody (Cell Signaling Technology, Danvers, Massachusetts, USA; Cat no. 8480), rabbit anti-phospho-GSK-3β (Ser9) antibody (Cell Signaling Technology, Danvers, Massachusetts, USA; Cat no. 5558), rabbit anti-GSK-3β antibody (Cell Signaling Technology, Danvers, Massachusetts, USA; Cat no. 12456). The blots were washed and incubated with the corresponding IRDye secondary antibody (LI-COR 800CW or 680RD anti-mouse/ anti-rabbit) in blocking buffer containing 0.01% Tween 20 for 1 hour at room temperature. The blots were washed and scanned using the infrared fluorescence detection Odyssey Imaging System (LI-COR Biosciences) to visualize the bound antibody. Housekeeping GAPDH signal was used for normalization of loading differences. Total GSK-3β signal was used for normalization of the level of phospho-GSK-3β (Ser9). For evaluation of the levels of YAP and β-catenin in the nuclear compartment by immunoblotting, parasite challenged and time matched control MHEC cells were washed in cold PBS and resuspended in hypotonic buffer (20 M Tris-HCl, pH 7.4, 25 mM HEPES, 10 mM NaCl, 3 mM MgCl_2_) containing protease inhibitor cocktail set III and phosphatase inhibitor cocktails 2 and 3 at 1:100 each followed by addition of 10% NP-40. The cells were vortexed centrifuged for 10 minutes at 3000 rpm at 4°C. Cytoplasmic extraction was done twice and the nuclear pellet was resuspended in the cell extraction buffer (20 M Tris-HCl, pH 7.4, 25 mM HEPES, 10 mM NaCl, 3 mM MgCl_2_) containing protease inhibitor cocktail set III and phosphatase inhibitor cocktails 2 and 3 at 1:100 each. The lysed nuclear fraction was centrifuged for 30 minutes at 14,000g at 4°C. The supernatant nuclear fraction was used in our assays. Nuclear lysates (20 μg/well) were separated by SDS-PAGE using 12% polyacrylamide gels and transferred onto nitrocellulose membranes. The membranes were blocked with Intercept TBS Blocking Buffer (LI-COR Biosciences) followed by incubation with the appropriate primary antibody (1:1000) at 4°C overnight on a shaker; rabbit anti-β-Catenin antibody (CST; Cat no. 8480), rabbit anti-GSK-3β antibody (CST; Cat no. 12456), mouse anti-HDAC (CST; Cat no. 5356) and mouse anti-GAPDH monoclonal antibody (SCBT; Cat no sc-47724). The blots were washed and incubated with the corresponding IRDye secondary antibody (LI-COR 800CW or 680RD anti-mouse/ anti-rabbit) in blocking buffer containing 0.01% Tween 20 for 1 hour at room temperature and scanned using the Odyssey Imaging System (LI-COR Biosciences) to visualize the bound antibody. The HDAC signal was used for normalization of loading differences and GAPDH signal was used to check for cytoplasmic contamination of the nuclear extract. Each experiment was done in biological triplicates and quantitation of band intensity was performed by densitometry using Image J. Statistical analysis of each parameter for the *T*. *cruzi* treated groups compared with non-treated control groups (mock infected groups, 0 h) using student’s *t*-test or one-way ANOVA (non-parametric) with Newman-Keuls post-hoc test. The difference was considered statistically significant if *P< 0.05, **P< 0.01 and ***P< 0.001 vs control.

### Immunofluorescence assays

MHEC were seeded on glass coverslip coated with 1% gelatin in 6-well culture plate. *T*. *cruzi* trypomastigotes Tulahuen strain clone MMC 20A (10 parasites per cell) were incubated with the MHEC for different times 1, 2, 3 and 6 h. The parasites were washed off with DPBS. The cells were fixed with 4% paraformaldehyde for 5 min at room temperature and washed with 1x DPBS. Fixed cells were perforated with 0.1% Triton-X100 in TBS for 5 min and blocked with 3% BSA-PBS for 30 min at room temperature. For evaluation of nuclear translocation, slides were incubated with rabbit anti-β-Catenin monoclonal antibody diluted 1:100 (Cell Signaling Technology, Danvers, Massachusetts, USA; Cat no. 8480) and phalloidin (1:2,000) in 1% BSA-PBS at 4°C overnight. The cells were washed with 1% BSA-PBS and re-probed with goat anti rabbit IgG secondary antibody conjugated with Alexa flour 647 (1:1,000) in wash buffer for 1 h. They were further washed and mounted with mounting medium containing DAPI (Life Technologies) to stain the nuclei. For colocalization assays, the fixed, perforated and blocked cells on the slides were incubated with a mixture of antibodies containing mouse anti-YAP monoclonal antibody diluted 1:100 (Santa Cruz Biotechnology cat no. sc-271134), rabbit anti-β-Catenin (D10A8) monoclonal antibody (Cell signaling Technology, cat no. 8480) diluted1:100, and phalloidin (1:2,000) at 4°C overnight. The cells were washed and the bound primary antibodies were detected using the corresponding secondary antibodies cocktail; goat anti-mouse Alexa fluor 488 and goat anti-rabbit Alexa fluor 647 antibodies diluted 1:1,000 in 1% BSA-PBS for 1 h at room temperature, washed and mounted with mounting media containing DAPI to stain the nuclei. The coverslips with treated cells were transferred on to glass slides and sealed. Stained slides were analyzed using the Nikon A1R confocal microscope located at the Centralized Core Facility at Meharry Medical College. Cellular and nuclear intensities including Pearson’s correlation coefficient for colocalization of signals were determined by imaging software NIS Elements AR Analysis version 5.20.02 64-bit.

### Wnt/β-catenin pathway inhibition and infection assays

To evaluate the importance of Wnt signaling pathway in the process of *T*. *cruzi* infection, we preincubated MHEC in the presence or absence of 20 μM *endo*-IWR1 inhibitor, challenged them with GFP–*T*. *cruzi* and evaluated the level of cellular infection by reading the fluorescence intensity of GFP parasites in the cells with a fluorimeter and confocal microscopy assays. For the fluorometric assays, WT MHEC monolayers growing on 1% gelatin precoated 48-well plates were pretreated with or without 20 μM of *endo*-IWR1 inhibitor for 72 hours. The preincubated WT MHEC were challenged with GFP- *T*. *cruzi* parasites at a ratio of 10 parasites per cell. The challenged cells received fresh complete medium daily for 72 h. At the end of 72 h, the cells were washed with 1x DPBS (without calcium/magnesium) and the GFP intensity was read. Fluorescence intensities were recorded at an emission of 485/20 and excitation of 528/20 using a microplate reader (BioTek SYNERGY, SOTWARE Gen5 version 2.05) and compared to similarly treated control cells not challenged with the transgenic parasites. Each biological replicate was prepared in triplicates. TSP1 KO MHEC challenged with GFP–*T*. *cruzi* in the absence of the inhibitor were treated similarly. The average mean fluorescence intensity of infected MHEC replicates with or without inhibitor treatment compared to their respective uninfected controls were calculated and plotted using the graphpad prism software package.

For the fluorescence microscopy assays, WT MHEC seeded on 6 well plastic culture plates coated with 1% gelatin were pretreated with or without 20 μM *endo*-IWR1 for 72 h. The cells were then challenged with GFP–*T*. *cruzi* at a ratio of 10 parasites per cells and fed with fresh complete medium daily for 72 h. The cells were washed with 1x DPBS (without calcium/magnesium). Live cells were stained with NucBlue Live ReadyProbes Reagent CellMask, Orange Actin Tracking Stain as described by the manufacturer (Thermofisher) and visualized using the Keyence All-in-One Fluorescence Microscope BZ-X710 and analyzed with BZ-X Analyzer software located at the Centralized Core Facility at Meharry Medical College.

### Statistical analysis

Data from at least three biological independent experiments are expressed as mean ± SEM. Statistical comparisons were made between controls and *T*. *cruzi* treated groups. Statistical analysis was done using Student’s *t*-test or one-way analysis of variance (ANOVA) for multiple groups of data followed by a Newman-Keuls test. P-value strength increases with number of asterisks P<0.05 (*), P<0.01 (**), and P<0.001 (***). Statistical analyses were performed using GraphPad Prism (GraphPad Software, San Diego, CA, USA).

## Results

### The levels of Wnt-5a ligand expressed in heart endothelial cells during the early phase of experimental *T*. *cruzi* infection is dependent on TSP1

**Fig 1:**
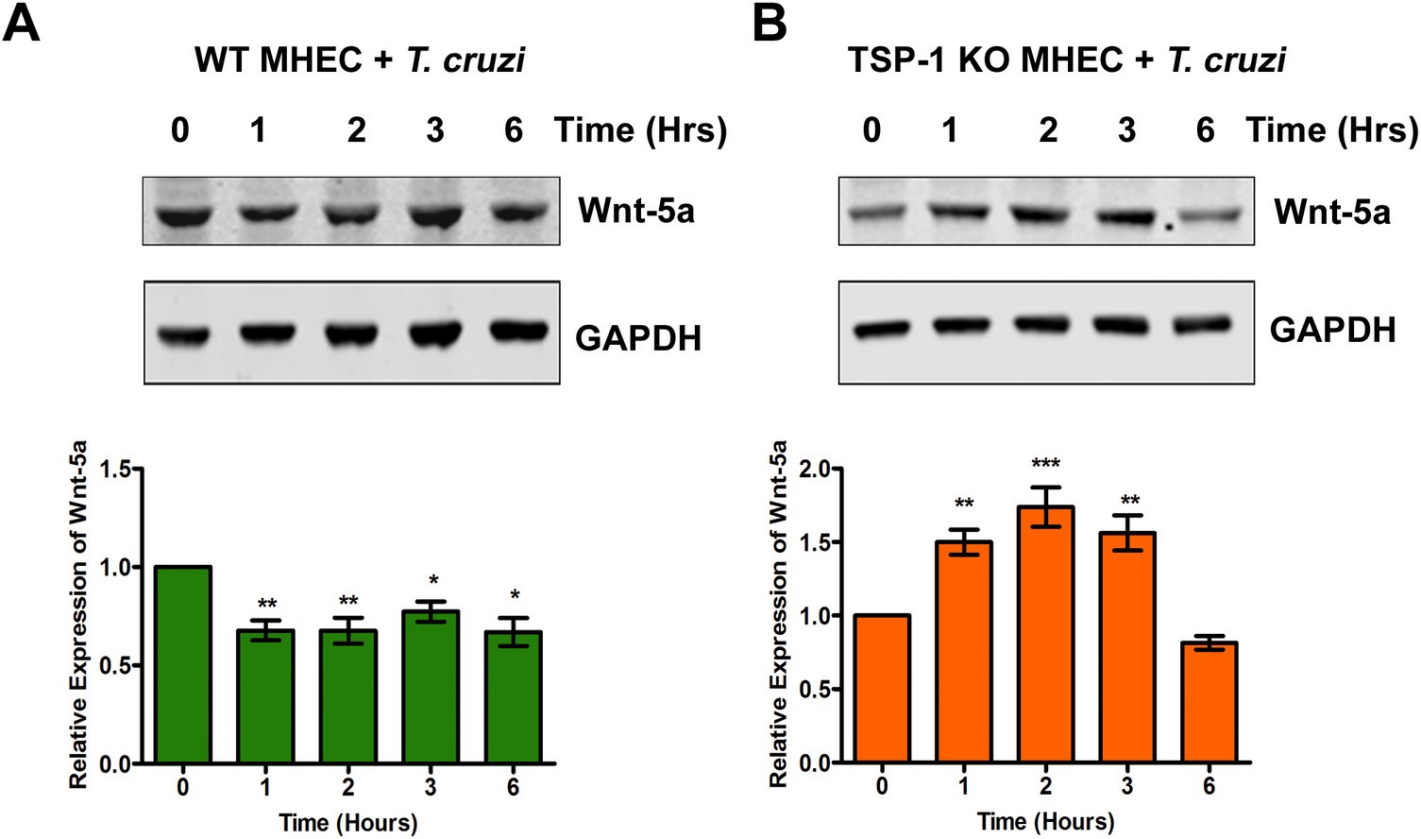
Experimental *T*. *cruzi* infection induces the expression of Wnt-5a in heart endothelial cells. Lysates (20 μg) from WT or TSP1 KO MHEC challenged with *T*. *cruzi* at different time points were resolved by SDS-PAGE, blotted, and probed with antibodies against Wnt-5a antibody (A) WT MHEC and (B) TSP 1KO MHEC and developed as described. The blots were stripped, reprobed with antibodies against housekeeping GAPDH and developed with the corresponding IRDye conjugated secondary antibody. The developed blots were scanned using the infrared fluorescence detection Odyssey Imaging System. The normalized fold change of Wnt-5a at each time point was determined and plotted in the bar graph for WT MHEC (A, lower panel) and for TSP1 KO MHEC (B, lower panel), respectively. The bar graphs represent mean values ± SE from three independent biological replicates. The value of P< 0.05 was considered significant. *P*<* 0.05; **P*<* 0.01; ***P*<* 0.001.

We showed that host TSP1 plays an essential role in cellular infection by *T*. *cruzi* and parasite induced pathogenesis. Others have suggested that the Wnt signal transduction cascade also plays important roles in cardiac pathophysiology. We next determined the importance of TSP1 expression and regulation of Wnt/β-catenin signaling proteins and their associated signaling cascade during the early phase of *T*. *cruzi* infection. We challenged WT and TSP1 KO MHEC with *T*. *cruzi* for different lengths of time (0, 1, 2, 3 and 6 h) and determined the levels of Wnt-5a by immunoblot assays. We found that when WT MHEC were challenged with *T*. *cruzi*, the levels of Wnt-5a protein decreased significantly at 1 h (0.67±0.05; P< 0.01), and 2 h (0.67±0.06; P< 0.01) compared with control. The significant decrease in the levels of Wnt-5a remained almost constant at 3 h (0.77±0.05; P< 0.05) and at 6 h (0.66±0.07; P< 0.05) time points compared with control (Fig 1A). In contrast, when TSP1 KO MHEC (in the absence of TSP1) were challenged with the parasite, the levels of Wnt-5a showed a continuous increase from 1 h (1.49±0.08; P< 0.01) to a maximum at 2 h (1.73±0.13; P< 0.001). The Wnt-5a level remained high at 3 h (1.56±0.11; P< 0.01) before showing a decrease at 6 h compared with control (Fig 1B). These results indicate that the Wnt ligand, Wnt-5a, was significantly increased in the TSP1 deficient MHEC challenged with *T*. *cruzi*.

### GSK-3β is phosphorylated at serine 9 in heart endothelial cells during the early phase of *T*. *cruzi* infection

**Fig 2:**
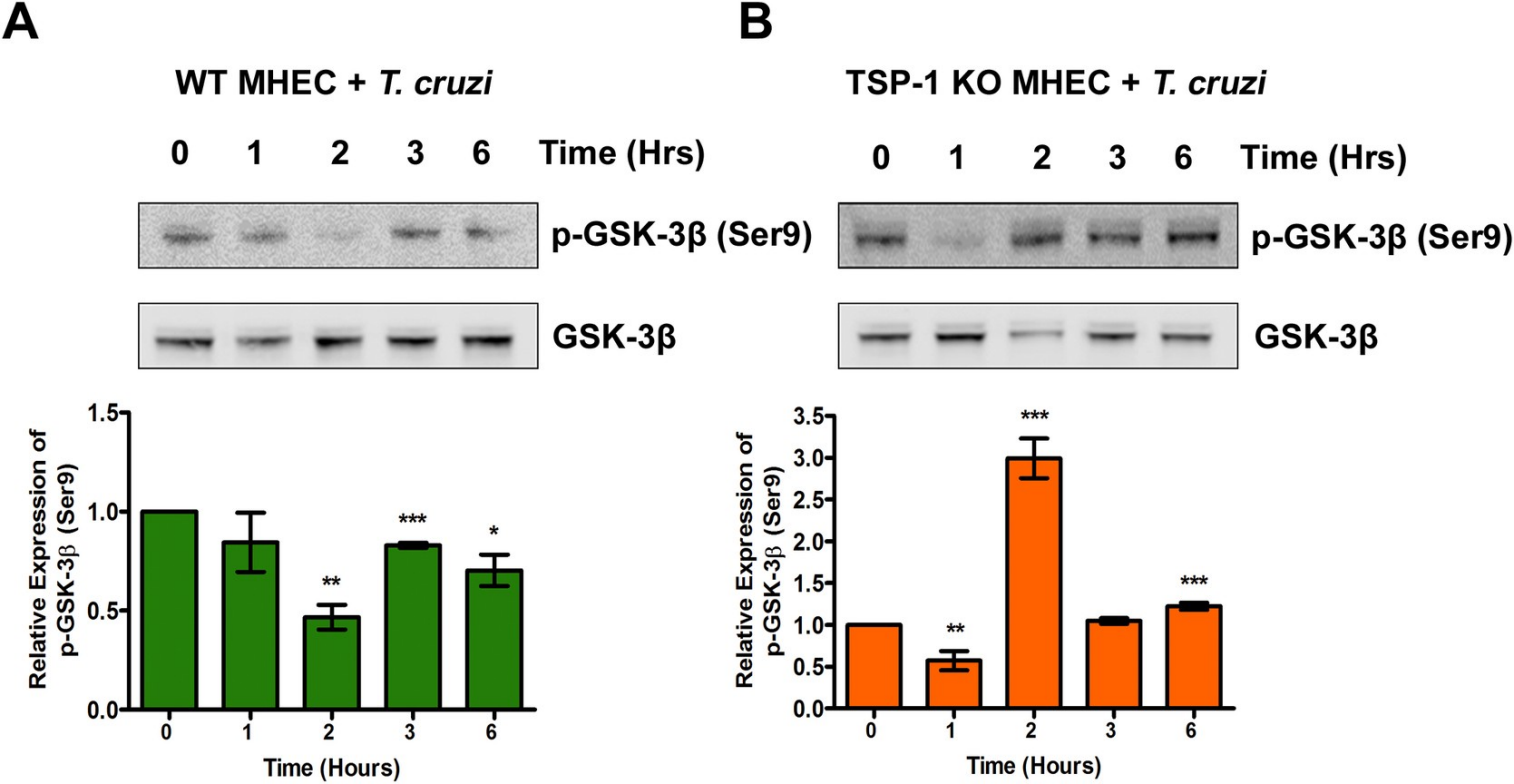
*T*. *cruzi* infection induces the phosphorylation of GSK-3β at Serine 9 in heart endothelial cells. Lysates (20 μg) from WT or TSP1 KO MHEC challenged with *T*. *cruzi* at different time points were resolved by SDS-PAGE, blotted, and probed with antibodies against p-GSK-3β (Ser9) and GSK-3β in (A) WT MHEC (B) TSP1 KO MHEC, respectively and developed with the corresponding IRDye conjugated secondary antibody. The developed blots were scanned using the infrared fluorescence detection Odyssey Imaging System. The normalized fold change in the levels of p-GSK-3β were determined and plotted in the bar graph for (A) WT MHEC, and (B) TSP1 KO MHEC, respectively. The bar graphs represent mean values ± SE from three independent biological replicates. The value of P< 0.05 was considered significant. *P*<* 0.05; **P*<* 0.01; ***P*<* 0.001.

We found that *T*. *cruzi* infection resulted in a significant increase in the levels of Wnt-5a in TSP1 KO MHEC. When the Wnt ligand is increased, it binds to its receptor and induces dissociation of the destruction complex in a series of phosphorylation events, which includes the phosphorylation of GSK-3β at serine 9. To evaluate if the increase in Wnt-5a ligand leads to an increase in the levels of phosphorylated GSK-3β (Ser9) during the early phase of *T*. *cruzi* infection, we challenged WT and TSP1 KO MHEC with *T*. *cruzi* for different times (0, 1, 2, 3 and 6 h) and analyzed the levels of phospho-GSK-3β (Ser9) using immunoblot assays. We found that when WT MHEC were challenged with *T*. *cruzi*, the levels of p-GSK-3β showed a continuous decrease from 1 h (0.84±0.14) to a minimum at 2 h (0.47±0.06; P< 0.01) followed with an increase at 3 h (0.83±0.01; P<0.001) and 6 h (0.7±0.08; P< 0.05) compared with control, which all lower than the control (Fig 2A).

When TSP1 KO MHEC (in the absence of TSP1) were challenged with the parasite, we observed a significant decrease in the levels of p-GSK-3β (Ser9) at 1 h (0.57±0.12; P< 0.01) followed by an increasing trend from 2- to 6 h that was maximum at 2 h (2.99±0.24; P< 0.001) compared with control (Fig 2B). These results indicate that the GSK-3β, a component of the destruction complex, is inactivated by phosphorylation, therefore, turning on the Wnt/β-catenin signaling in TSP1 KO MHEC challenged with *T*. *cruzi*.

### Experimental *T*. *cruzi* infection induces β-catenin stabilization during the early phase of cellular infection

**Fig 3:**
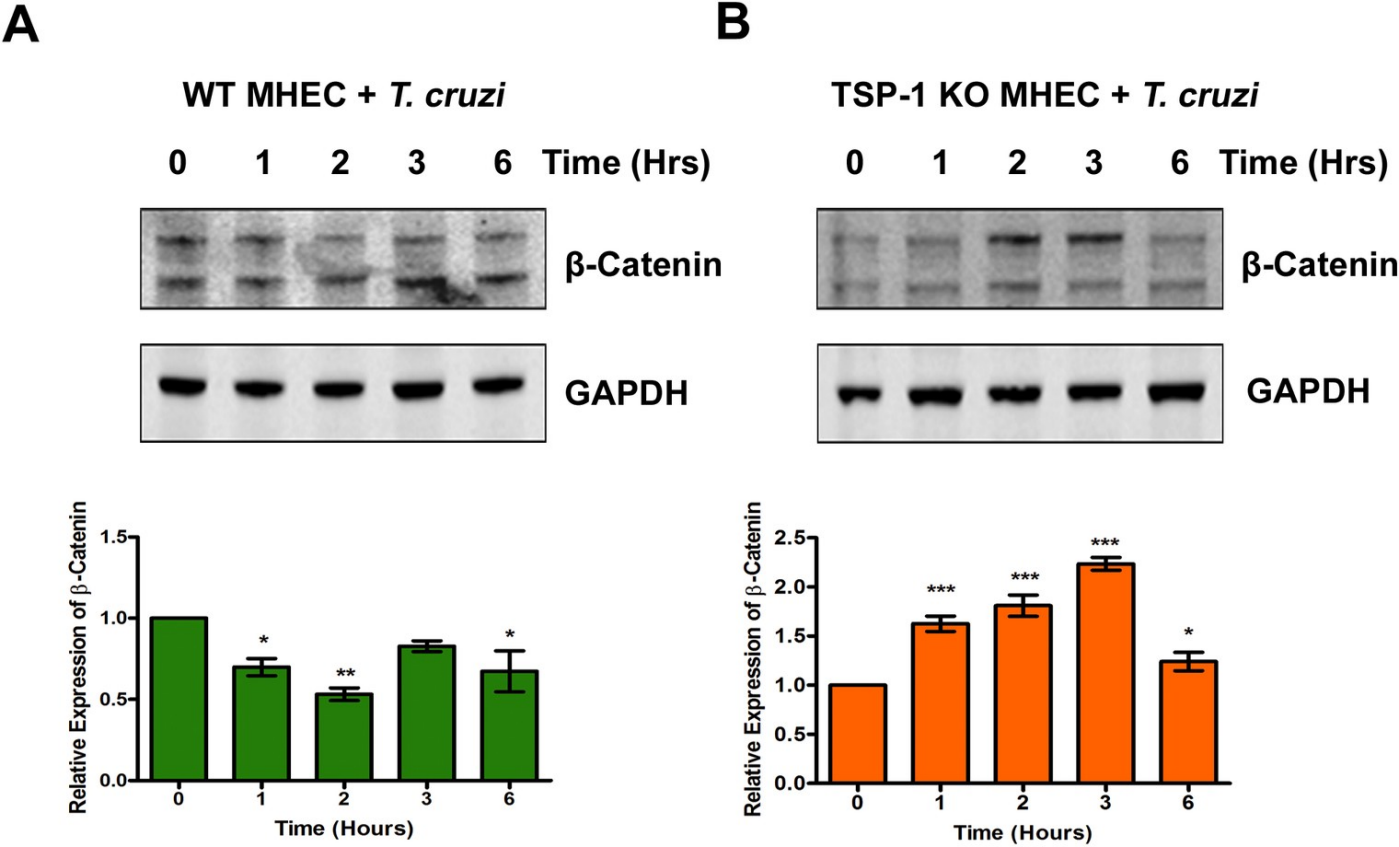
*T*. *cruzi* induces the β-catenin stabilization in the absence of TSP1 in heart endothelial cells. Lysates (20 μg) from WT or TSP1 KO MHEC challenged with *T*. *cruzi* at different time points were resolved by SDS-PAGE, blotted, and probed with antibodies against β-catenin in (A) WT MHEC and (B) TSP1 KO MHEC and developed as described. The blots were stripped, reprobed with antibodies against housekeeping GAPDH and developed with the corresponding IRDye conjugated secondary antibody. The developed blots were scanned using the infrared fluorescence detection Odyssey Imaging System. The normalized fold changes in the level of β-catenin were determined and plotted in the bar graph for WT MHEC (A, lower panel) TSP1 KO MHEC (B, lower panel), respectively. The bar graphs represent mean values ± SE from three independent biological replicates. The value of P< 0.05 was considered significant. *P*<* 0.05; **P*<* 0.01; ***P*<* 0.001.

Our data showed that *T*. *cruzi* significantly increased the levels of Wnt-5a and p-GSK-3β in the absence of TSP1. Phosphorylation of GSK-3β, a component of the destruction complex in the presence of the Wnt signaling ligand, leads to the stabilization of β-catenin. We next determined whether increased levels of Wnt-5a and p-GSK-3β lead to an increase in the level of stable β-catenin during the early phase of *T*. *cruzi* infection. We challenged WT and TSP1 KO MHEC with *T*. *cruzi* for different lengths of time (0, 1, 2, 3 and 6 h) and evaluated the levels of stable β-catenin using immunoblot assays. When WT MHEC were challenged with *T*. *cruzi*, the levels of unphosphorylated β-catenin showed a general decreasing trend from 1 h (0.69±0.05; P< 0.05), to a minimum at 2 h (0.53±0.03; P< 0.01), followed by a gradual increase which was significant at 6 h (0.67±0.12; P< 0.05) compared with control (Fig 3A). In contrast, when TSP1 KO MHEC were challenged with the parasite the levels of unphosphorylated stable β-catenin showed a continuous increase at all time points. From 1 h (1.62±0.07; P< 0.001), 2 h (1.80±0.10; P< 0.001) to a maximum at 3 h (2.23±0.06; P< 0.001) and then to 6 h (1.24±0.09; P< 0.05) compared with control (Fig 3B). These results indicated that the Wnt/β-catenin signaling pathway is turned ‘on’ leading to significantly higher amounts of β-catenin stabilization in TSP1 KO MHEC challenged with *T*. *cruzi*.

### Nuclear translocation of β-catenin during the early phase of *T*. *cruzi* infection is dysregulated by TSP1

**Fig 4:**
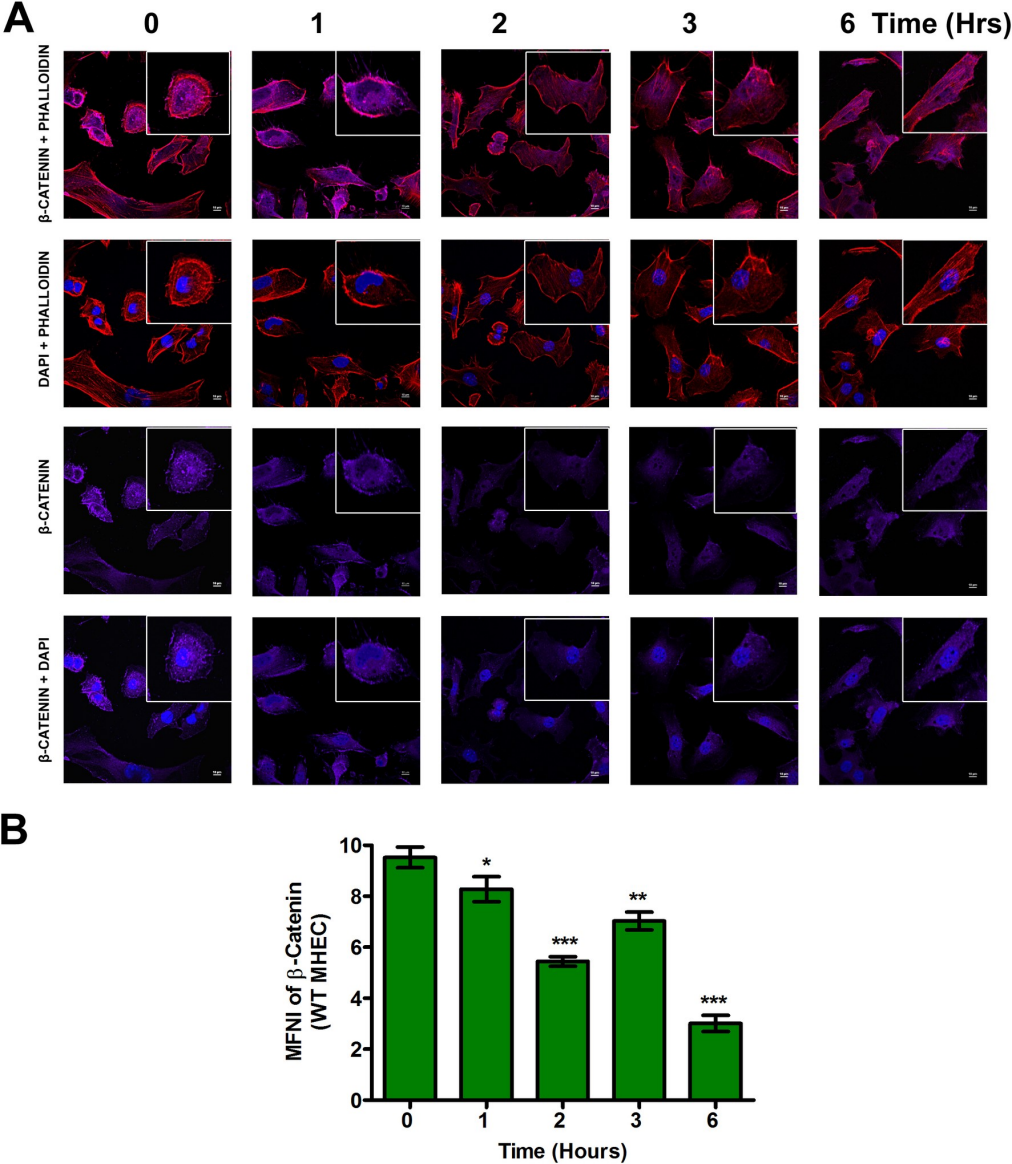
Nuclear translocation of β-catenin in WT MHEC decreases during early phase of *T*. *cruzi* infection. WT MHEC grown on glass coverslips coated with 1% gelatin in 6-well culture plates were challenged with *T*. *cruzi* at different time points, washed, fixed, perforated with 0.1% Triton-X100, blocked with 3% BSA-PBS, and incubated at 4°C overnight in solutions containing phalloidin and (A) rabbit anti β-catenin antibodies. The cells were washed and reprobed with goat anti rabbit Alexa flour 647 conjugated secondary antibody. The washed cells were mounted with mounting media containing DAPI to stain the nuclei. (B) Stained slides were analyzed by confocal microscopy and the mean nuclear fluorescence intensity (MNFI) values were plotted for β-catenin. Images were capture at 60X at scale bar 10 μm. Each confocal microscopy image is a representative of three independent biological replicates. The bar graphs represent MNFI values ± SE from three independent biological replicates. The value of P*<* 0.05 was considered significant. *P*<* 0.05; **P*<* 0.01; ***P*<* 0.001.

**Fig 5:**
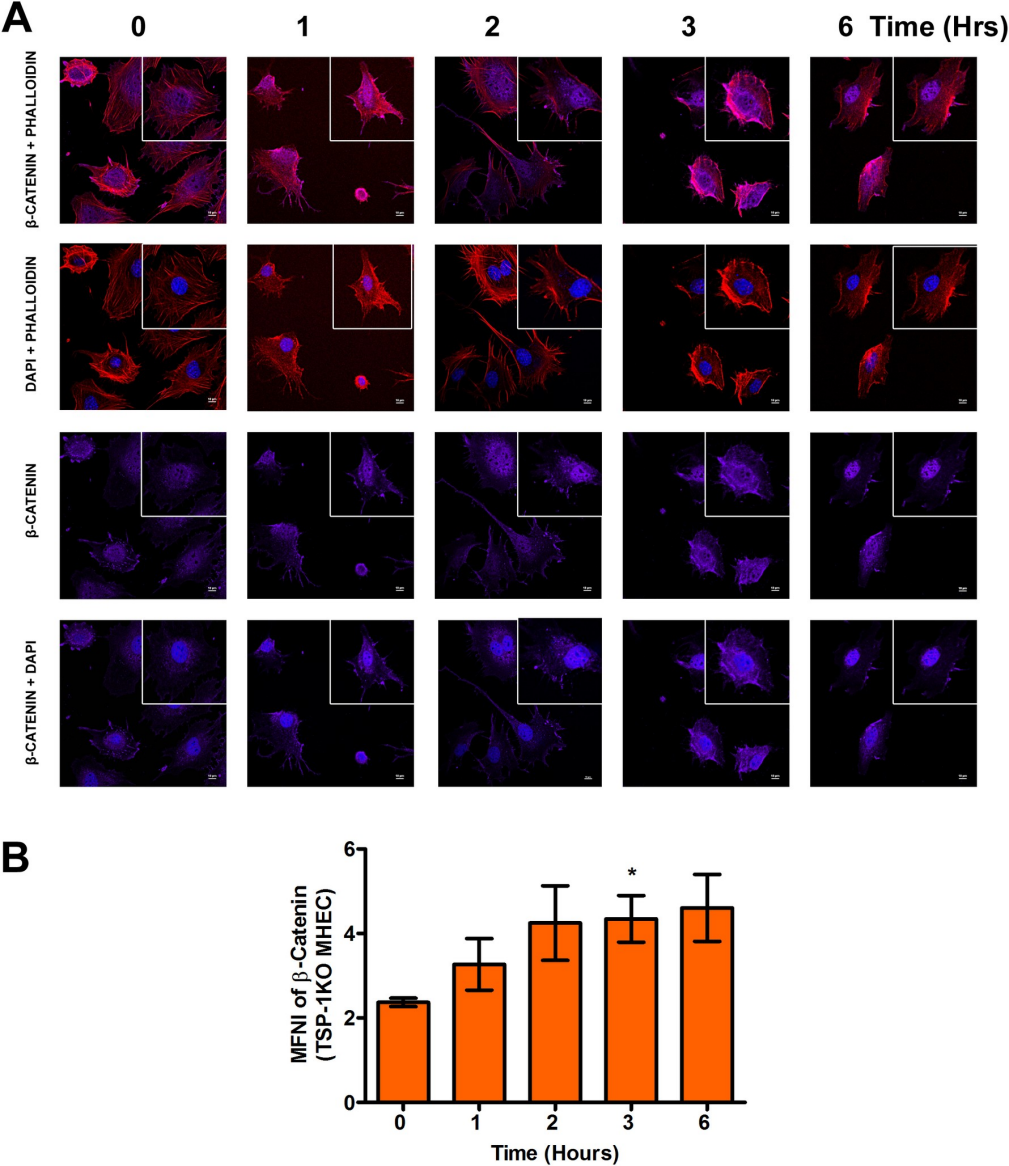
*T*. *cruzi* infection induces nuclear translocation of β-catenin in TSP1 deficient heart endothelial cells. TSP1 KO MHEC grown on glass coverslips coated with 1% gelatin in 6-well culture plates were challenged with *T*. *cruzi* at different time points, washed, fixed, perforated with 0.1% Triton-X100, blocked with 3% BSA-PBS, and incubated at 4°C overnight in solutions containing phalloidin and (A) rabbit anti β-catenin antibodies. The cells were washed and reprobed with goat anti rabbit Alexa flour 647 conjugated secondary antibody. The washed cells were mounted with mounting media containing DAPI to stain the nuclei. (B) Stained slides were analyzed by confocal microscopy and the mean nuclear fluorescence intensity (MNFI) values were plotted for β-catenin. Images were capture at 60X at scale bar 10 μm. Each confocal microscopy image is a representative of three independent biological replicates. The bar graphs represent MNFI values ± SE from three independent biological replicates. The value of P*<* 0.05 was considered significant. *P*<* 0.05; **P*<* 0.01; ***P*<* 0.001.

We found that *T*. *cruzi* infection induced significant changes in the levels of stabilized β-catenin in TSP1 KO MHEC during the early phases of infection. Stabilized β-catenin translocates into the nucleus to promote the expression of target genes. Next, we investigated whether increased levels of β-catenin correspond to an increase in nuclear β-catenin during the early phase of *T*. *cruzi* infection. We challenged WT and TSP1 KO MHEC with *T*. *cruzi* for different lengths of time (0, 1, 2, 3 and 6 h) and evaluated the levels of nuclear translocation of the downstream Wnt effector molecule β-catenin using confocal microscopy. Our data show that when WT MHEC were challenged with *T*. *cruzi* trypomastigotes, the level of β-catenin nuclear translocation significantly decreased at all time points from 1–6 h with a sinusoidal characteristic as shown by the decrease in mean nuclear fluorescence intensity of the fluorophore in several fields. The mean nuclear fluorescence intensity of β-catenin showed a significant decrease from 1 h (8.28±0.49; P< 0.05); 2 h (5.44±0.18; P< 0.001) and continued to 3 h (7.02±0.35; P< 0.01) and finally 6 h (3.01±0.31; P< 0.001) compared with control (Fig 4A and 4B). In contrast, when TSP1 KO MHEC were challenged with the parasite the mean fluorescence intensity of β-catenin translocated into the nuclear compartment showed a significant gradual increase from 1–6 h, which plateaued from 2–6 h (Fig 5A and 5B). These results suggest that β-catenin is translocated into the nucleus during the early phase of *T*. *cruzi* infection especially in TSP1-deficient cells.

### Evaluation of the levels of β-catenin and YAP in nuclear fractions of MHEC during the early phase of *T*. *cruzi* challenge

**Fig 6:**
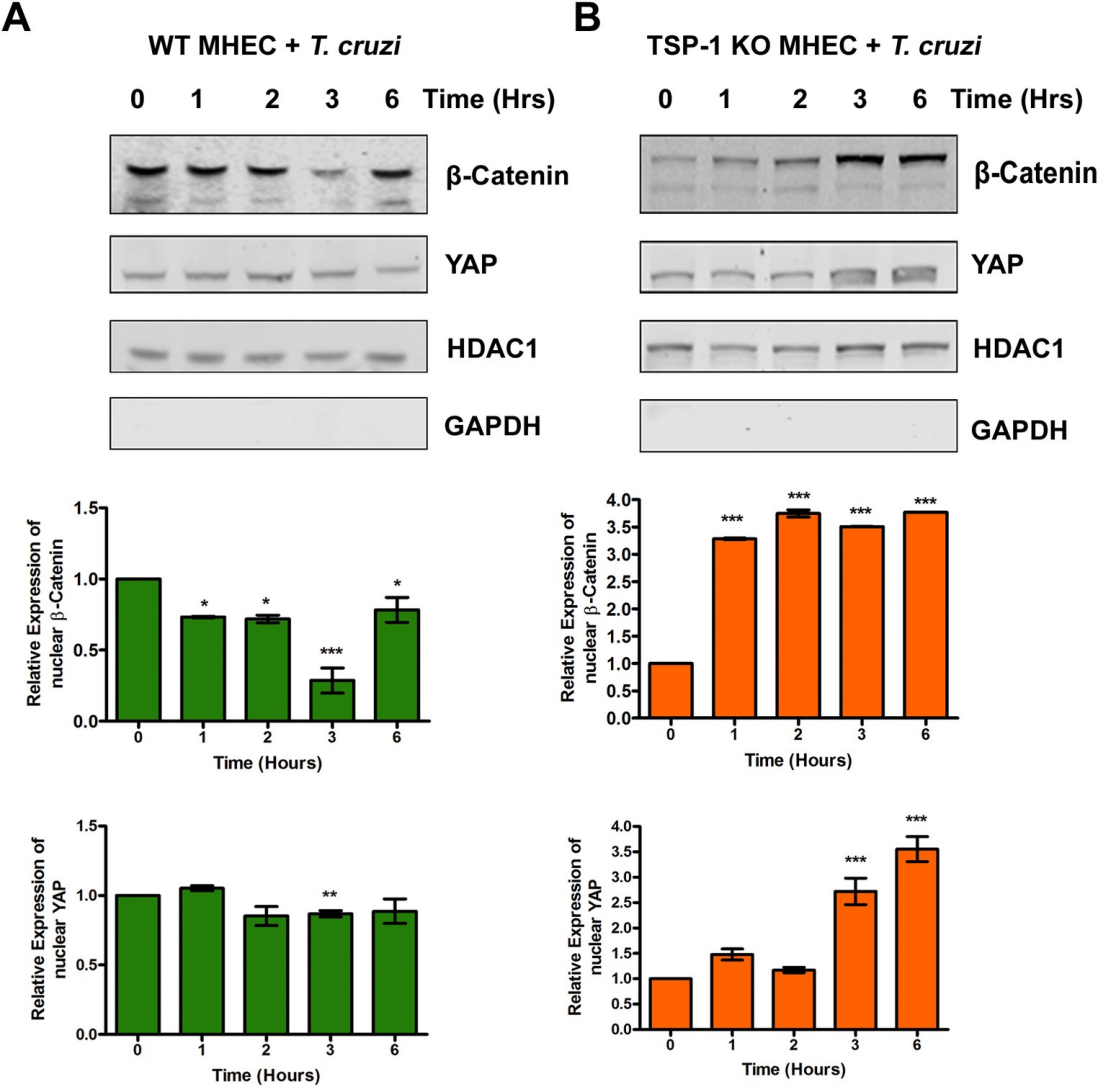
Levels of β-catenin and YAP in nuclear extracts of MHEC during the early phase of *T*. *cruzi* challenge. Nuclear fractions (20 μg) from WT or TSP1 KO MHEC challenged with *T*. *cruzi* at different time points were resolved by SDS-PAGE, blotted, and probed with antibodies against β-catenin or YAP in (A) WT MHEC and (B) TSP1 KO MHEC, respectively and developed as described. The blots were stripped, reprobed with antibodies against HDAC1 and developed with the corresponding IRDye conjugated secondary antibody. The blots were further probed with antibodies against GAPDH and developed with the corresponding IRDye conjugated secondary antibody. The developed blots were scanned using the infrared fluorescence detection Odyssey Imaging System. The HDAC1 normalized fold changes in the level of β-catenin and YAP were determined and plotted in the bar graph for WT MHEC (A, middle and lower panel), TSP1 KO MHEC (B, middle and lower panel), respectively. The bar graphs represent mean values ± SE from three independent biological replicates. The value of P< 0.05 was considered significant. *P*<* 0.05; **P*<* 0.01; ***P*<* 0.001.

To complement our confocal immunofluorescence microscopy assays, we extracted the nuclear fractions from parasite challenged WT, TSP1 KO MHEC, and their respective time matched controls and evaluated the levels of β-catenin and YAP. We observed that when WT MHEC cells were challenged with *T*. *cruzi*, the level of β-catenin in nuclear extracts decreased at all time points from 1 h (0.73±0.01; p<0.05) and continuously to a minimum at 3 h (0.29±0.09; p<0.001) before increasing at 6 h (0.78±0.09; p<0.05) showing a sinusoidal characteristic. The relative levels of nuclear YAP decreased to a significant minimum at 3 h (0.87±0.02; p<0.01) and remained low compared with the control (Fig 6A; upper and lower panels). The relative levels of β-catenin and YAP in the nuclear extracts of time matched WT MHEC not challenged with the parasite did not show any significant change compared with control (S1A Fig; upper and lower panels). In contrast, the nuclear extract levels of β-catenin in TSP1 KO MHEC challenged with the parasite increased significantly at 1 h (3.28±0.01; p<0.001) and remained continuously significantly high to 6 h (3.77±0.001; p<0.001). The relative levels of nuclear YAP showed a continuous increase which became significant at 3 h (2.72±0.26; p<0.001) and 6 h (3.55±0.25; p<0.001) compared with control (Fig 6B; upper and lower panels). The relative levels of β-catenin and YAP in the nuclear extracts of time matched TSP1 KO MHEC not challenged with the parasite did not show any significant change compared with control (S1B Fig; upper and lower panels). Our data indicate that during the early phase of *T*. *cruzi* infection of heart endothelial cells, nuclear levels of β-catenin and YAP are significantly increased in the absence of TSP-1.

### β-catenin and YAP colocalize in the nucleus of heart endothelial cells during the early phase of *T*. *cruzi* infection

**Fig 7:**
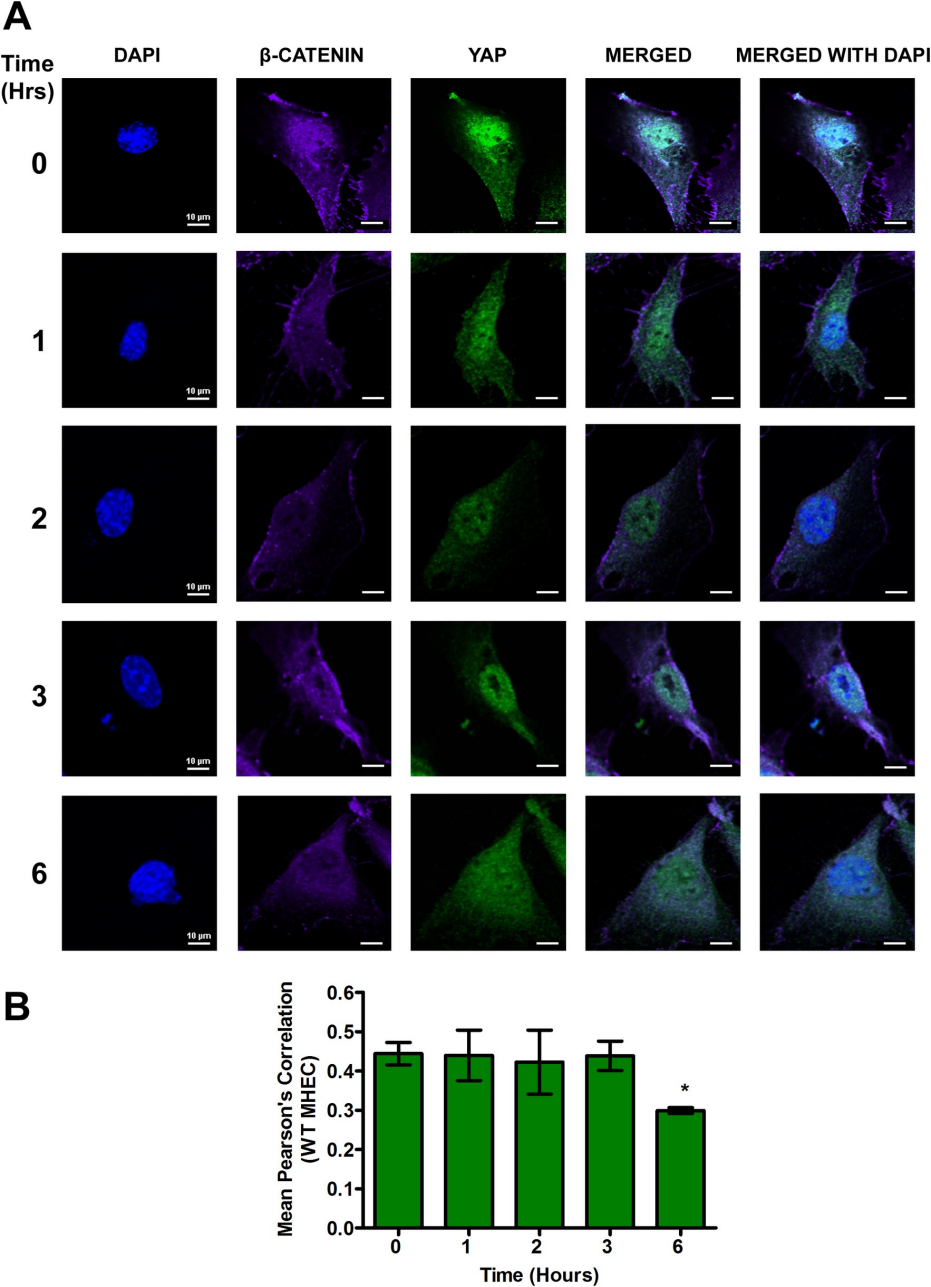
Nuclear translocation of β-catenin/YAP during early phase *T*. *cruzi* infection is steady in WT MHEC. WT MHEC grown on glass coverslips coated with 1% gelatin in 6-well culture plates were challenged with *T*. *cruzi* at multiple time points, washed, fixed, perforated with 0.1% Triton-X100, blocked with 3% BSA-PBS, and incubated at 4°C overnight in solutions containing phalloidin and (A) mouse anti YAP and rabbit anti β-catenin antibodies. The washed cells were reprobed with a cocktail of goat anti mouse Alexa flour 488 and goat anti rabbit Alexa flour 647 conjugated secondary antibodies. The cells were washed and mounted with mounting media containing DAPI to stain the nuclei. Stained slides were analyzed by confocal microscopy and images were captured at 60X at scale bar 10 μm. The mean fluorescence intensities of the merged signals were analyzed using confocal microscopy software to generate Pearson’s correlation coefficients. (B) The bar graphs represent Pearson’s correlation coefficients values ± SE from three independent biological replicates. Each confocal microscopy image is a representative of three independent biological replicates. The value of P*<* 0.05 was considered significant. *P*<* 0.05; **P*<* 0.01; ***P*<* 0.001.

**Fig 8:**
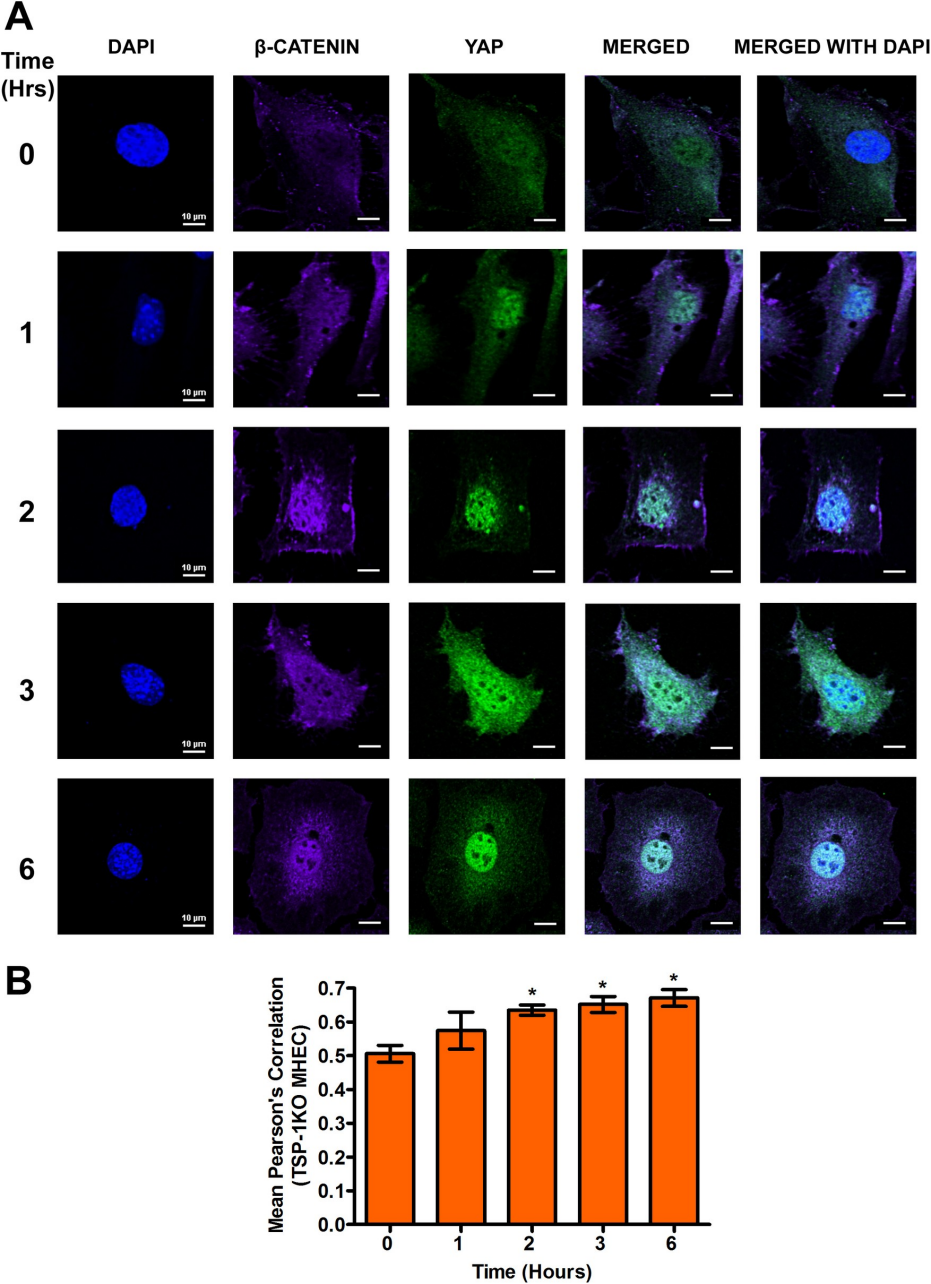
Experimental *T*. *cruzi* infection of TSP1 KO MHEC induces continuous increase in β-catenin/YAP nuclear colocalization. TSP1 KO MHEC grown on glass coverslips coated with 1% gelatin in 6-well culture plates were challenged with *T*. *cruzi* at multiple time points, washed, fixed, perforated with 0.1% Triton-X100, blocked with 3% BSA-PBS, and incubated at 4°C overnight in solutions containing phalloidin and (A) mouse anti YAP and rabbit anti β-catenin antibodies. The washed cells were reprobed with a cocktail of goat anti mouse Alexa flour 488 and goat anti rabbit Alexa flour 647 conjugated secondary antibodies. The cells were washed and mounted with mounting media containing DAPI to stain the nuclei. Stained slides were analyzed by confocal microscopy and images were captured at 60X at scale bar 10 μm. The mean fluorescence intensities of the merged signals were analyzed using confocal microscopy software to generate Pearson’s correlation coefficient values. (B) The bar graphs represent Pearson’s correlation coefficients values ± SE from three independent biological replicates. Each confocal microscopy image is a representative of three independent biological replicates. The value of P*<* 0.05 was considered significant. *P*<* 0.05; **P*<* 0.01; ***P*<* 0.001.

We recently reported that TSP1 plays an essential role in YAP nuclear translocation during the early phase of *T*. *cruzi* infection of heart endothelial cells. To evaluate whether the translocated YAP in the nucleus co-localizes with the β-catenin noted here, we challenged WT and TSP1 KO MHEC with *T*. *cruzi* for different times (0, 1, 2, 3 and 6 h). We determined the levels of nuclear co-localization of the downstream Wnt effector molecule, β-catenin, with YAP by measuring the mean Pearson’s correlation coefficient of both proteins in the nucleus using confocal microscopy. In the presence of TSP1 in WT MHEC, we observed that the mean Pearson’s correlation coefficient of β-catenin and YAP nuclear colocalization remained steady from 1–3 h before showing a significant decrease at 6 h (0.29±0.007; P< 0.05) compared with control (Fig 7A and 7B). However, in the absence of TSP1, TSP1 KO MHEC, we observed a gradual increase in the nuclear colocalization of β-catenin and YAP. This was demonstrated by a significant gradual increase in the mean Pearson’s correlation coefficient from 2 h (0.63±0.01; P< 0.05); 3 h (0.65±0.02; P< 0.05) and a maximum at 6 h (0.67±0.02; P< 0.05) compared with the control (Fig 8A and 8B). These results suggest that during the early phase of *T*. *cruzi* infection of heart endothelial cells, there is potentially a crosstalk between the Wnt and hippo signaling pathways.

### Inhibition of Wnt signaling pathway with *endo*-IWR 1 significantly decreases cellular infection by *T*. *cruzi*

**Fig 9:**
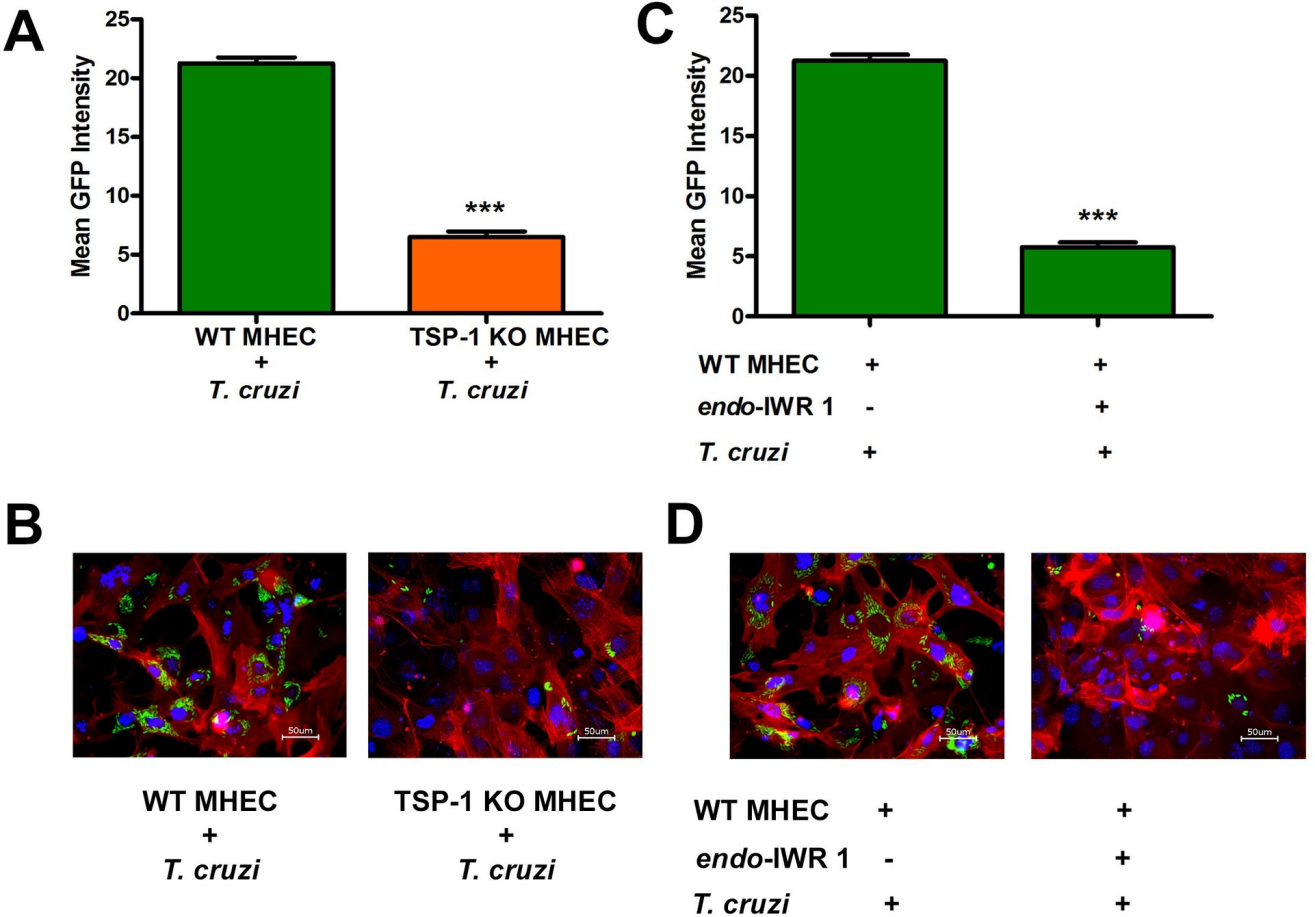
Inhibition of Wnt signaling pathway or absence of TSP1 decreases cellular *T*. *cruzi* infection. (A) WT MHEC and TSP1 KO MHEC seeded in 1% gelatin coated 48-well plates were challenged with transgenic GFP-expressing *T*. *cruzi* trypomastigotes, fed with fresh complete media daily for 72 h. Infected and control cells were washed with 1X DPBS and GFP fluorescence intensities were recorded, emission 485/20 and excitation 528/20 using microplate reader (BioTek SYNERGY, SOTWARE Gen5 version 2.05). The mean fluorescence intensities of each replicate compared to control were computed and plotted. (B) WT MHEC and TSP1 KO MHEC were seeded on 1% gelatin in 6-well culture plates and challenged with the transgenic GFP-expressing *T*. *cruzi* trypomastigotes. Cells were fed with fresh complete media daily for 72 h. NucBlue Live ReadyProbes Reagent was used to stain the nuclei and CellMask Orange Actin Tracking Stain was used to stain actin. The stained cells were visualized using the Keyence All-in-One Fluorescence Microscope BZ-X710 and analyzed with BZ-X Analyzer software. (C) WT MHEC and TSP1 KO MHEC seeded in 1% gelatin coated 48-well plates were preincubated with or without 20 μM *endo*-IWR1 for 72 h and challenged with transgenic GFP-expressing *T*. *cruzi* trypomastigotes for 72 h with daily medium change. Infected and control cells were washed with 1X DPBS and GFP fluorescence intensities were recorded, emission 485/20 and excitation 528/20 using microplate reader (BioTek SYNERGY, SOTWARE Gen5 version 2.05). The mean fluorescence intensities of each replicate compared to control were computed and plotted. (D) WT MHEC and TSP1 KO MHEC seeded on 1% gelatin coated 6-well plates were preincubated with or without 20 μM *endo*-IWR1 for 72 h and challenged with transgenic GFP-expressing *T*. *cruzi* trypomastigotes for 72 h with daily media change. NucBlue Live ReadyProbes Reagent was used to stain the nuclei and CellMask Orange Actin Tracking Stain was used to stain actin. The stained cells were visualized using the Keyence All-in-One Fluorescence Microscope BZ-X710 and analyzed with BZ-X Analyzer software. The value of P*<* 0.05 was considered significant. *P*<* 0.05; **P*<* 0.01; ***P*<* 0.001.

We observed that during the early phase of *T*. *cruzi* infection of heart endothelial cells, there is nuclear translocation and colocalization of both YAP and β-catenin. This suggests that the crosstalk between these pathways plays an important role during cellular infection by *T*. *cruzi*. We also showed that TSP1 plays an essential role during *T*. *cruzi* infection and pathogenesis. To evaluate whether disruption of the YAP and β-catenin colocalization will have an effect on *T*. *cruzi* infection, we pre-incubated WT MHEC with 20 μM *endo*-IWR 1 for 72 h, reported to inhibit the Wnt signaling pathway, and evaluated cellular infection of the pretreated cells with GFP-*T*. *cruzi* using fluorimetry to evaluate GFP intensity and confocal microscopy to show parasite infectivity and density. We observed that the mean GFP intensity of WT MHEC infected with the transgenic parasites expressing GFP was 21.3±0.5 compared with that of TSP1 KO MHEC (6.5±0.45; P< 0.001), which were significantly less infected (Fig 9A). Additionally, microscopy images showed that TSP1 KO MHEC are significantly less infected (Fig 9B). When WT MHEC were pretreated with *endo*-IWR 1 before the challenge with transgenic parasites expressing GFP, we observed that the mean GFP intensity of the infected pretreated WT MHEC (7.5±0.25; P< 0.001) was significantly less than WT MHEC (23.75±0.58), supported by the microscopy data (Fig 9C and 9D). The MHEC model used in our assays are successfully infected by *T*. *cruzi* (S2 Fig and S1 Movie). These results indicate that inhibition of the Wnt signaling pathway could significantly decrease cellular infection of WT MHEC *in vitro*.

## Discussion

Chagas disease caused by the parasite *T*. *cruzi* is a major cause of cardiac pathology in approximately 30% of infected individuals. The parasite infects all nucleated cells of the body including endothelial cells [2]. Invasive trypomastigotes released from infected cells are carried in the blood to infect cells and organs that are nearby or distant from site of parasite release. During the process of transportation, the parasites can signal, interact and infect heart endothelial cells causing disease. Endothelial cells are very important in the process of *T*. *cruzi* infection and pathogenesis because parasites are evident in these cells before parasitemia can be detected implying that coronary endothelial cells constitute the primary or initial target of *T*. *cruzi* infection [51]. Despite the significance of endothelial cells in the pathogenesis of *T*. *cruzi* infection, the molecular mechanisms by which the parasite signal and infect heart endothelial cells remain to be completely characterized despite ongoing research. Elucidation of the molecular interaction between *T*. *cruzi* and heart endothelial cells can be exploited for the development of novel molecular intervention strategies.

We showed that during the early phase of infection, *T*. *cruzi* induces host cells to upregulate TSP1 expression both at the transcript and protein levels. Increased expression of host TSP1 is essential for infection since downregulation of TSP1 expression by RNAi or use of inhibitors that reduce TSP1 expression significantly abrogates the host cell infection with the parasite [21]. Additionally, in an *ex vivo* model using mouse embryo fibroblasts (MEF), we showed that MEF generated from TSP1 KO mice were significantly less infected compared to MEF from WT mice supporting the importance of TSP1 in the process of cellular infection [22]. The upregulation of TSP1 by the parasite during cellular infection activates transforming growth factor beta (TGFβ), which has been suggested to play important roles in *T*. *cruzi* infection and cardiovascular health including the functioning of vascular cells like endothelial cells [52,53]. Furthermore, we recently showed that the parasite regulates expression of PIWI-interacting RNA (piRNA) that can target TGFβ [54]. Elucidation of the host cells signaling mechanisms dysregulated by increased TSP1 expression to enhance cellular infection and pathogenesis remains an important aspect of *T*. *cruzi* research.

We recently showed that TSP1 plays an important role in the dysregulation of the hippo signaling pathway during the early phase of *T*. *cruzi* infection [55]. In addition, it was suggested that the Wnt/β-catenin pathway is activated early after *T*. *cruzi* infection of bone marrow derived macrophages [56]. However, the significance of TSP1 expression in activation of the Wnt/β-catenin pathway, including the possibility of molecular interaction between YAP and β-catenin, during the early phase of infection remains unknown. Thus, we hypothesize that during the early phase of molecular interaction between *T*. *cruzi* and heart endothelial cells, TSP1 plays an important role in the nuclear crosstalk between the Hippo effector molecule, YAP and the Wnt effector molecule, β-catenin, and that disruption of this suggested crosstalk will affect parasite cellular infectivity.

To investigate the significance of TSP1 expression in the molecular colocalization between YAP and β-catenin during the early phase of cellular infection, and how this colocalization affects parasite infection of heart endothelial cells *in vitro*, we challenged MHEC from WT and TSP1 KO mice with invasive *T*. *cruzi* trypomastigotes Tulahuen strain clone MMC20A for different times. We evaluated the activation of Wnt/β-catenin pathway, the molecular colocalization of Wnt/Hippo signaling pathway effector molecules, and how its disruption could affect the early phase of *T*. *cruzi* infection. Our data showed that the parasite turns on the Wnt signaling pathway in MHEC in a TSP1 dependent manner. Specifically, in the absence of TSP1, the levels of the Wnt ligand, Wnt-5a, which activates the Wnt signaling pathway was significantly increased at 1, 2 and 3 h time points while it remained toned down and steady in the presence of TSP1 (Fig 1A and 1B). Thus, TSP1 expression may suppress Wnt signaling and as a result enhance infection.

Our observations agree with others who suggested that the parasite can activate Wnt proteins during the early phase of cellular infection [56]. Additionally, we showed that the degree of activation of Wnt-5a is affected by TSP1 expression during the early phase of infection. Furthermore, we showed that the parasite significantly increased the levels of p-GSK-3β and accordingly significantly decreased the level of GSK-3β post challenge in TSP1 KO MHEC compared with an opposite observation in WT MHEC (Fig 2A and 2B). Our observations support the contemporary knowledge which suggests that Wnt ligands bind to Fzd receptors to activate the Wnt signaling pathway by inhibition of GSK-3β activity through its phosphorylation which disrupts the formation of the β-catenin destruction complex [27,29,31]. In agreement with the significant activation of Wnt signaling cascade proteins and the deactivation of the destruction complex (Figs 1 and 2), the level of β-catenin in TSP1 KO MHEC lysate showed a significant increase following parasite challenge (Fig 3B) compared with control. In WT MHEC, the level of β-catenin decreased to a minimum at 2 h followed by a gradual increase (Fig 3A).

Our data agrees with others who showed that destabilization of the destruction complex limits β-catenin phosphorylation, ubiquitination and proteasomal degradation with a consequent accumulation of the protein in the cytoplasm [34,57,58]. Furthermore, our data showed that the increase in β-catenin levels in the absence of TSP1 was accompanied by an increase in the nuclear translocation of β-catenin in TSP1 KO MHEC, where we observed a continuous increase in nuclear β-catenin (Fig 5A and 5B). In contrast, in WT MHEC we observed a significant decrease in nuclear translocation of β-catenin (Fig 4A and 4B). This immunofluorescence microscopy observations were supported by immunoblotting assays showing increased levels of β-catenin and YAP in nuclear extracts of parasite challenged TSP1 KO MHEC (Fig 6B). This increase was specifically induced by the parasite challenge since time matched controls showed no significant changes in the levels of β-catenin or YAP in WT or TSP1 KO MHEC, respectively (S1 Fig).

Others also reported that when the Wnt signaling pathway is turned on, the downstream effector molecule, β-catenin is translocated into the nucleus [29,59]. Others suggested that fold change rather than absolute β-catenin levels are critical indicating that low levels of nuclear β-catenin translocation would be sufficient for initiation of transcriptional changes [60]. Experimental models showed that overexpression and nuclear accumulation of β-catenin triggered a fibrogenic response, however, β-catenin depletion in the same model did not abrogate a fibrogenic response indicating that the Wnt signaling pathway functions in collaboration with other signaling pathways to induce a fibrogenic response [58,61]. Since we showed that TSP1 dysregulates YAP nuclear translocation during the early phase of infection, we evaluated whether Wnt/β-catenin and YAP molecules colocalize in the nuclear compartment during the early phase of infection especially as TSP1 is important for *T*. *cruzi* infection and pathogenesis. Our current studies evaluating the nuclear colocalization of β-catenin and YAP during the early phase of *T*. *cruzi* infection, opened up new studies in our laboratory geared towards deciphering the molecular mechanisms operating in the presence and absence of TSP1.

Our data revealed, for the first time, in the context of *T*. *cruzi* research that in the presence of TSP1 (WT MHEC), the parasite induced nuclear colocalization of β-catenin and YAP (Fig 7A and 7B), while in the absence TSP1 (TSP1 KO MHEC) the level of β-catenin/YAP nuclear colocalization showed a gradual increase from 2 to 6 h (Fig 8A and 8B). Immunoblotting assays using nuclear extracts of parasite challenged MHEC showed the presence of both molecules (Fig 6). Others have suggested the existence of a complex crosstalk between YAP and β-catenin in several systems where YAP regulated β-catenin activity and β-catenin regulated YAP/TAZ activity, and they have been suggested to be involved in the pathogenesis of tissue fibrosis and tumorigenesis [32,33,45,57,58]. Considering our data showing that the Pearson’s correlation coefficient of YAP/β-catenin nuclear colocalization is significantly increased in the absence of TSP1, supported by data showing increasing amounts of both molecules in nuclear extracts (Fig 7B), coupled with our previous report that *T*. *cruzi* infection *ex vivo* and *in vitro* is significantly reduced in the absence of TSP1, or when TSP1 is knocked down by RNAi, respectively [21,22], we preincubated WT MHEC with *endo*-IWR 1, an inhibitor of Wnt signaling and evaluated *T*. *cruzi* infectivity. Our data showed that inhibition of the Wnt signaling pathway significantly reduced *T*. *cruzi* infection of WT MHEC. The level of infection of the WT MHEC pretreated with *endo*-IWR 1 was reduced to that of *T*. *cruzi* infection of TSP1 KO MHEC. A decrease in the level of infection in the inhibitor treated cells also led to a decrease in parasite density in the infected cells (Fig 9A–9D). Others showed that inhibition of the Wnt signaling pathway decreased parasite multiplication [56]. In that study, it was not clear if *T*. *cruzi* infectivity was similar in cells pretreated or not with Wnt pathway inhibitors. We and others now show that inhibition of the Wnt/β-catenin signaling pathway significantly decreased parasite infection and multiplication. These results suggest that the Wnt pathway inhibition has a potential preventative therapy for *T*. *cruzi* infection. Therefore, this pathway can be exploited for the development of specific molecular intervention strategy for the management of *T*. *cruzi* infection.

## Supporting information

S1 Fig The nuclear levels of β-catenin and YAP remain unchanged in time matched controls. (TIF)

S2 Fig MHEC sustained mature *T. cruzi* infection followed by invasive trypomastigotes parasite release. (TIF)

S1 Movie Video showing release of mature invasive *T. cruzi* trypomastigotes from infected MHEC. (MOV)
