# Supplementary figures and images for: Thrombospondin-1 expression and modulation of Wnt and hippo signaling pathways during the early phase of Trypanosoma cruzi infection of heart endothelial cells
Source: PLoS Negl Trop Dis. 2022 Jan 5;16(1):e0010074. doi: 10.1371/journal.pntd.0010074 (PMC8730400; doi:10.1371/journal.pntd.0010074)

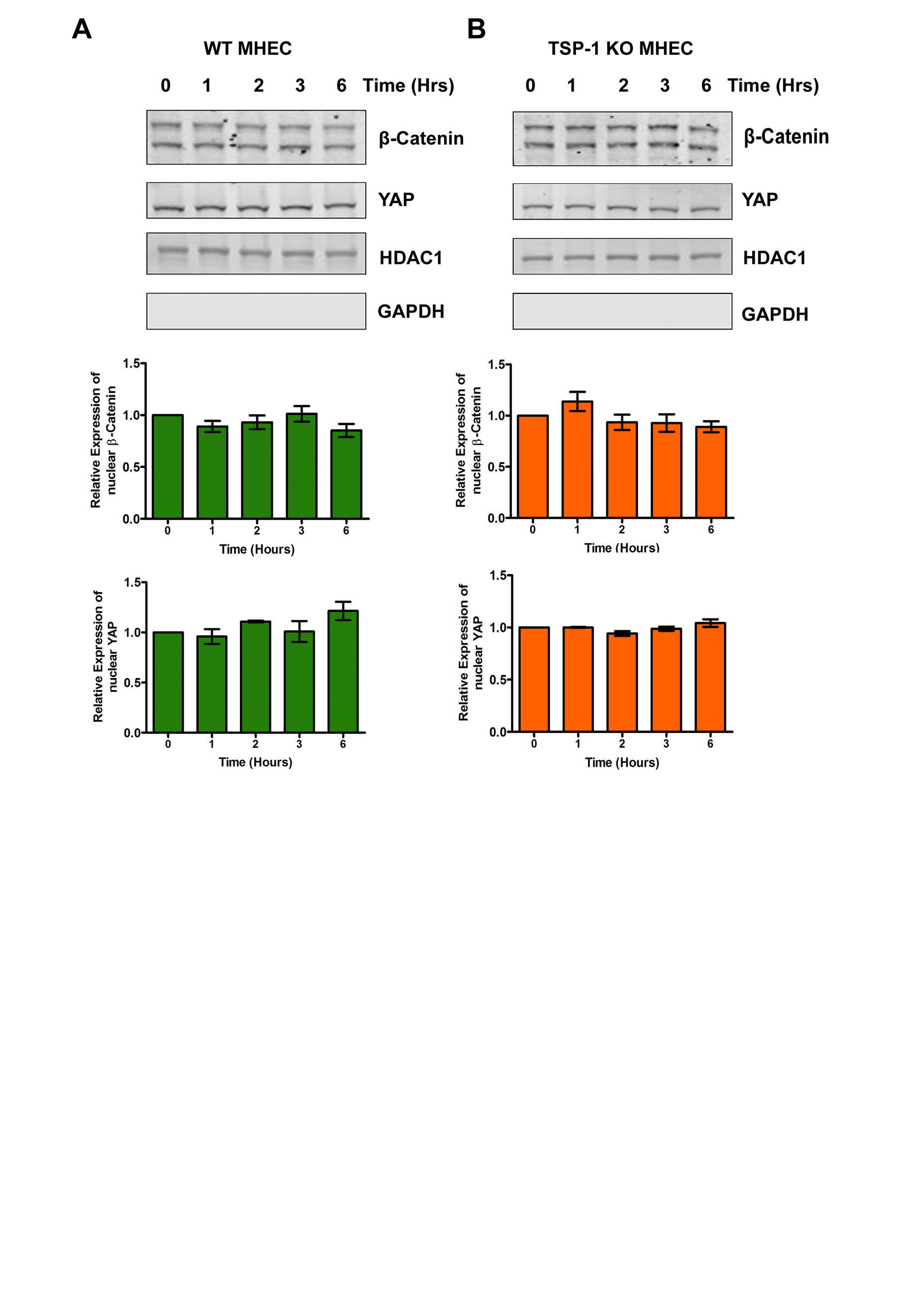

Supplement: S1 Fig — (TIF) [file pntd.0010074.s001.tif]

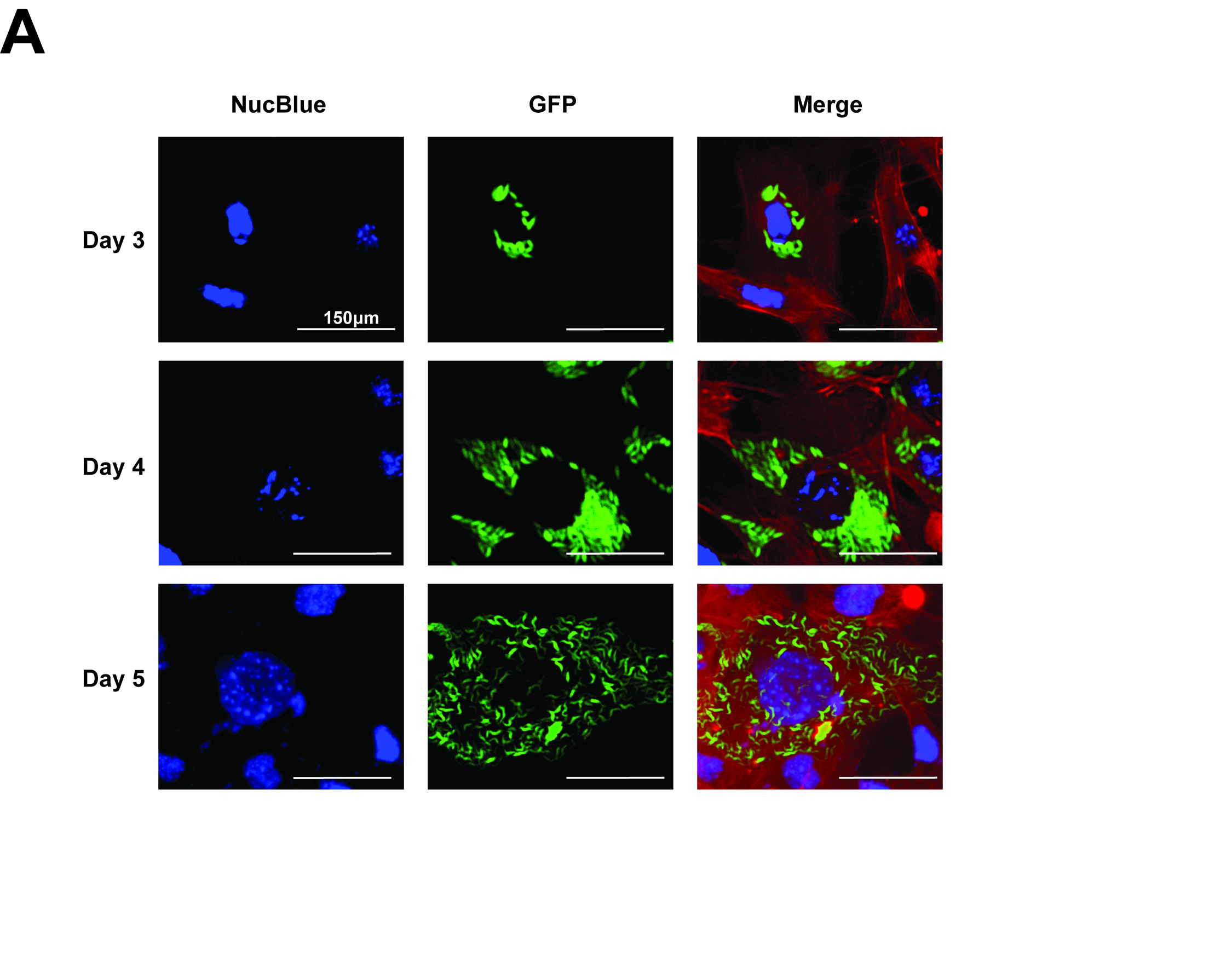

Supplement: S2 Fig — (TIF) [file pntd.0010074.s002.tif]
